# Genome assembly of *Ottelia alismoides*, a multiple-carbon utilisation aquatic plant

**DOI:** 10.1186/s12863-024-01230-0

**Published:** 2024-05-23

**Authors:** Zheng-Feng Wang, Lin-Fang Wu, Lei Chen, Wei-Guang Zhu, En-Ping Yu, Feng-Xia Xu, Hong-Lin Cao

**Affiliations:** 1 Key Laboratory of Vegetation Restoration and Management of Degraded Ecosystems, South China Botanical Garden, Chinese Academy of Sciences, Guangzhou, 510650 China; 2 Key Laboratory of National Forestry and Grassland Administration on Plant Conservation and Utilization in Southern China, South China Botanical Garden, Chinese Academy of Sciences, Guangzhou, 510650 China; 3 South China National Botanical Garden, Guangzhou, 510650 China; 4 Guangzhou Linfang Ecological Technology Co., Ltd, Guangzhou, 510000 China; 5 University of Chinese Academy of Sciences, Beijing, 100049 China

**Keywords:** de novo assembly, genome feature, genome survey, gene annotation, next generation seqencing, RNA-seq

## Abstract

**Objectives:**

*Ottelia* Pers. is in the Hydrocharitaceae family. Species in the genus are aquatic, and China is their centre of origin in Asia. *Ottelia alismoides* (L.) Pers., which is distributed worldwide, is a distinguishing element in China, while other species of this genus are endemic to China. However, *O. alismoides* is also considered endangered due to habitat loss and pollution in some Asian countries. *Ottelia alismoides* is the only submerged macrophyte that contains three carbon dioxide-concentrating mechanisms, i.e. bicarbonate (HCO_3_^−^) use, crassulacean acid metabolism and the C4 pathway. In this study, we present its first genome assembly to help illustrate the various carbon metabolism mechanisms and to enable genetic conservation in the future.

**Data description:**

Using DNA and RNA extracted from one *O. alismoides* leaf, this work produced ∼ 73.4 Gb HiFi reads, ∼ 126.4 Gb whole genome sequencing short reads and ∼ 21.9 Gb RNA-seq reads. The *de novo* genome assembly was 6,455,939,835 bp in length, with 11,923 scaffolds/contigs and an N50 of 790,733 bp. Genome assembly completeness assessment with Benchmarking Universal Single-Copy Orthologs revealed a score of 94.4%. The repetitive sequence in the assembly was 4,875,817,144 bp (75.5%). A total of 116,176 genes were predicted. The protein sequences were functionally annotated against multiple databases, facilitating comparative genomic analysis.

## Objective

*Ottelia* Pers., an aquatic plant genus that includes approximately 24 extant species, is the second largest genus in the family Hydrocharitaceae [1, 2]. China is the centre for *Ottelia* in Asia. There are 10 *Ottelia* species in China, all of which are endemic, except *O. alismoides* [1, 2]. *Ottelia alismoides* (L.) Pers. is an annual or perennial herb that can be submersed or floating in fresh or salt water [1–4]. It is distributed worldwide, including Africa, Australia and Asia [4]. Molecular phylogeny analysis indicates that *O. alismoides* is the ancestor of the other *Ottelia* species in China [1, 2]. Due to the loss and deterioration of aquatic habitats due to anthropogenic activities, it has been endangered in both China and Japan [2, 4]. However, it is listed as a noxious weed in America [5]. One particular property in *O. alismoides* is that it is the only submerged macrophyte that contains three carbon dioxide-concentrating mechanisms, i.e. bicarbonate (HCO3^−^) use, crassulacean acid metabolism (CAM) and the C_4_ pathway [6, 7]. It can be used to treat water pollution [3, 8] and has as medicinal value, such as cancer and tuberculosis treatment [3, 9]. Therefore, our work provides a draft genome of *O. alismoides* to help depict the genetic bases of its different carbon usages and metabolism related to variable biochemical medicines for its conservation, management and utility in the future.

## Data description

Leaf samples from one *O. alismoides* individual planted in the South China Botanical Gard in Guangzhou, China, were collected. For genome assembly and annotation, three sequencing libraries were constructed using total RNA and genomic DNA extracted from the samples. Genomic DNA was extracted using the cetyltrimethylammonium bromide method, and total RNA was extracted using the TRNzol Universal RNA Extraction Kit (Tiangen, Beijing, China). The quality and quantity of DNA/RNA were assessed using the NanoDrop™ One microvolume UV-Vis Spectrophotometer (Thermo Fisher Scientific, California, USA) and gel electrophoresis. The PacBio Sequel II sequencer was used for circular consensus long read whole genomic sequencing (WGS), which is also known as HiFi sequencing. A MGI DNBSEQ-T7 sequencer was used for short-read WGS and RNA sequencing (RNA-seq), both under 150 bp paired-end mode. Using sequencing data, different programmes were applied to perform the analyses. In these analyses, the default parameters of the programmers were used unless otherwise mentioned.

The WGS short reads were trimmed with Sickle v1.33 [10] under the parameters of “-q 30 -l 80”. KmerGenie v1.7044 [11] was then used to estimate the *O. alismoides* genome size with the trimmed reads under the parameters of “-k 141 --diploid”. After removing adapters in HiFi reads by HiFiAdapterFilt v2.0.0 [12], hifiasm v0.19.6 [13] was used to assemble the *O. alismoides* genome. Duplicated sequences were further removed by Redundans 0.14a [14] and Purge_dups v1.2.5 [15]. Using RNA-seq data, the assembly was scaffolded with P_RNA_scaffolder [16], and the scaffolds were gap closed by TGS-GapClose 1.2.1 [17]. The completeness of the final assembly was assessed by BUSCO v5.7.0 [18] using the Embryphyta odb10 2020-09-10 database.

The assembly was parsed through RED v2.0 [19] and EDTA v2.1.0 [20] for repeat sequence identification. After combining the RED and EDTA results, the repeated sequences were then soft-masked in the assembly. Braker3 v.3.0.6 [21] was applied for initial gene prediction aided with transcriptome data and reference protein sequences (Data file 1) [22]. The braker results were then input into the Funannotate pipeline v1.8.16 [23] under the “funannotate prediction” command with the parameters “--max_intronlen 1000000”. The predicted genes were functionally annotated against multiple databases using the “funannotate annotate” command.

The sequencing libraries produced ∼ 73.4 Gb raw data for HiFi sequencing (Data file 2) [24], ∼ 126.4 Gb for WGS short read sequencing (Data file 3) [25] and ∼ 21.9 Gb for RNA-seq (Data file 4) [26]. The estimated genome size of *O. alismoides* was 6,863,432,158 bp, while the assembly was 6,455,939,835 bp with 11,923 scaffolds/contigs (N50 = 790,733 bp) (Data file 5) [27]. The BUSCO assessment indicated a completeness of 94.4% (Data file 6) [28]. EDTA and RED identified 3,695,203,717 bp (57.2%) (Data file 7) [29] and 4,138,710,098 bp (64.1%) (Data file 8) [30] of repetitive sequences, respectively, in the genome. Their combination was 4,875,817,144 bp, accounting for 75.5% of the genome (data file 9) [31]. A total of 116,176 genes were predicted (Data files 10–12) [32–34], and their annotation is shown in Data files 13 and 14 [35, 36].

## Limitations

The current assembled genome is still fragmented and could be further improved by increasing HiFi sequencing data and combining ultra-long Nanopore sequencing and Hi-C data.

**Table 1:** Overview of all data files/data sets

Label	Name of data file/data set	File types (file extension)	Data repository and identifier (DOI or accession number)
Data file 1	Table 1 Species with their protein sequences used for gene prediction	Table (.xlsx)	Figshare, 10.6084/m9.figshare.25498324.v1 [22]
Data file 2	HiFi reads	Fastq file (.fastq)	NCBI Sequence Read Archive, https://identifiers.org/ncbi/insdc.sra:SRR27887122 [24]
Data file 3	Raw WGS short reads	Fastq file (.fastq)	NCBI Sequence Read Archive, https://identifiers.org/ncbi/insdc.sra:SRR27887124 [25]
Data file 4	Raw RNA reads of leaf tissues	Fastq file (.fastq)	NCBI Sequence Read Archive, https://identifiers.org/ncbi/insdc.sra:SRR27887123 [26]
Data file 5	Assembled genome	Fasta file (.fasta)	NCBI Nucleotide, https://identifiers.org/nucleotide:JAZKJV000000000.1 [27]
Data file 6	BUSCO assessment of the assembly	Text (.txt)	Figshare, 10.6084/m9.figshare.25498345.v1 [28]
Data file 7	Repetitive sequences predicted by EDTA	Gff3 file (.gff3)	Figshare, 10.6084/m9.figshare.25499017.v1 [29]
Data file 8	Repetitive sequences predicted by RED	Text file (.bed)	Figshare, 10.6084/m9.figshare.25499059.v1 [30]
Data file 9	Repetitive sequences combined by RED and EDTA	Text file (.bed)	Figshare, 10.6084/m9.figshare.25499077.v1 [31]
Data file 10	Predicted gene	Gff3 file (.gff3)	Figshare, 10.6084/m9.figshare.25499086.v2 [32]
Data file 11	Predicted genes - nucleotide sequences	Fasta file (.fasta)	Figshare, 10.6084/m9.figshare.25499185.v1 [33]
Data file 12	Predicted genes - translated sequences	Fasta file (.fasta)	Figshare, 10.6084/m9.figshare.25499230.v1 [34]
Data file 13	Gene annotation using GO, Pfam, interPro and UniProt, dbCAN, MEROPS and SignalP databases	Text (.txt)	Figshare, 10.6084/m9.figshare.25499305.v1 [35]
Data file 14	Gene annotation from eggNOG-mapper analysis	Text (.txt)	Figshare, 10.6084/m9.figshare.25499254.v1 [36]

## Data Availability

Raw sequenced reads have been uploaded to the NCBI Sequence Read Archive under accession number SRR27887122 for HiFi reads, SRR27887124 for short-WGS sequencing reads, SRR27887123 for RNA-seq reads, and JAZKJV000000000 for the assembled genome. Please further see Table 1 in the manuscript for details and references of the results of the annotations submitted to figshare.
